# Tissue-specific changes in expression of *Vegfr2* in tumor and normal tissues of lymphoma-bearing BALB/c mice under chronic restraint stress

**DOI:** 10.1186/s13104-025-07219-x

**Published:** 2025-04-14

**Authors:** Alonso A. Orozco-Flores, Gloria Romero-Beltrán, Diana Caballero-Hernández, Deyanira Quistián-Martínez, Ricardo Gomez-Flores, Patricia Tamez-Guerra, Cristina Rodríguez-Padilla

**Affiliations:** 1 Universidad Autónoma de Nuevo León, Facultad de Ciencias Biológicas, Laboratorio de Inmunología y Virología, San Nicolás de los Garza, NL México; 2 Universidad Autónoma de Nuevo León, Facultad de Ciencias Biológicas, Departamento de Botánica, San Nicolás de los Garza, NL México

**Keywords:** Psychogenic stress, Cancer, Angiogenesis, VEGFR-2, Tumor, Fat, Brain

## Abstract

**Objective:**

Alteration of the expression of vascular endothelial growth factor (VEGF) and its receptors, VEGFR-1 and VEGFR-2, leads to aberrant angiogenesis in cancer; this is exacerbated by chronic stress. Our main aim was to determine the effect of chronic restraint stress on the expression of *Vegfr2*, the gene encoding VEGFR-2, in tumor, fat, skeletal muscle and brain in a murine model of lymphoma.

**Results:**

We found that both chronic stress and tumor burden alter *Vegfr2* expression. Under chronic stress, *Vegfr2* is differentially expressed in inguinal adipose tissue, decreasing in tumor-free, and increasing in tumor-bearing animals. In skeletal muscle, brain, and tumor, *Vegfr2* expression was upregulated by chronic stress. Adipose tissue, brain and skeletal muscle of tumor-bearing animals also showed changes in *Vegfr2* expression during tumor progression. We also found that for skeletal muscle the combination of chronic stress and tumor burden enhances *Vegfr2* expression (23-folds).

**Conclusion:**

Chronic stress and tumor burden influence *Vegfr2* expression in normal and tumoral tissues and their co-occurrence enhances its effect on skeletal muscle.

## Introduction

The stress of daily life is a significant public health concern, as it contributes to cancer progression by promoting specific capabilities, including angiogenesis. In lymphoma, increased tumor vascularization correlates with poor prognosis [1]. Increased tumor angiogenesis is mediated either by tumor-secreted factors such as vascular endothelial growth factor (VEGF) or by increasing expression of its receptors VEGFR-1 and VEGFR-2 [2]. VEGFR-2 has a critical role in angiogenesis in health and disease. In cancer, upregulation of *Vegfr2*, also found in the literature as *flk1*(mouse) and *Kdr* (human), has been linked to tumor progression in lung and gastric cancer in vitro, and its increased expression in tissues is considered a poor prognosis factor [3, 4]. This role in cancer is also supported by findings of impaired tumor growth and angiogenesis in heterozygous mice for *Vegfr2*/*Flk1*4 [5]. In addition, blocking of VEGFR-2 improves anti-tumor therapy in lung adenocarcinoma [6]. Several studies have shown alterations in the expression of *Vegf* and its receptors due to chronic psychogenic stress. *Vegfr2* expression is inhibited in the hippocampus of rats subjected to anger-induced stress [7]. In contrast, under chronic restraint stress, *Vegfr2* expression was shown to increase in mice’s prefrontal cortex [8]. In addition, chronic exposure to corticosterone, a stress hormone, decreases the expression of *Vegfr2*/*Flk1* in the prefrontal cortex of mice [9]. Mimicking stress with isoprenaline, an agonist of the β2-adrenergic receptor, upregulates *Vegfr2* in HUVEC and MGC803 cells from a tumor xenograft in BALB/c mice, an effect that was dependent of plexinA1/VEGFR2-JAK2-STAT3 activation [10, 11]. Thus, based on previous reports of altered *Vegfr2* under stress, and preliminary findings in female mice bearing the L5178Y-R lymphoma (unpublished), we hypothesized that chronic stress has the potential to modulate *Vegfr2* expression in tumor and normal tissues of lymphoma-bearing mice. In addition, we also hypothesize that tumor burden may affect the expression of *Vegfr2* in response to chronic stress by disrupting the host response to stressors. In the present study, we assessed the effect of chronic restraint stress and tumor burden on *Vegfr2* expression in tumor and normal tissues in the L5178Y-R murine lymphoma model.

## Main text

### Methods

#### Ethical statement

Experiments in the present study complied with the Mexican regulation NOM-062-ZOO-1999, related to Technical Specifications for the Production, Care, and Use of Laboratory Animals. The study was also reviewed and approved by the Institutional Committee for Research Ethics and Animal Welfare of Biological Sciences College at Autonomous University of Nuevo Leon (CEIBA-**2018-028**).

### Animals

Female BALB/c mice, aged 10–12 weeks, were obtained from the institucional animal facility and housed in groups of four per cage. Mice were supplied with free access to food and water. Cardboard and plastic tubes were added to cages to provide environmental enrichment. At 9 weeks of age, mice were randomly assigned to one of four experimental groups as follows: tumor-free mice, under resting (*n* = 3) and stress (*n* = 4) conditions, and tumor-bearing mice under resting (*n* = 4) and stress (*n* = 4) conditions.

### L5178Y-R tumor model

The L5178Y-R lymphoma is a well stablished, transplantable tumor model. For tumor implantation, 2 × 10^6^ L517Y-R cells (ATCC CRL-1722) in 0.2 mL of PBS were administered subcutaneously in the upper right thigh of mice, where a solid tumor subsequently developed. Tumor-free animals received 0.2 mL of PBS only. Mice were monitored daily for changes in body condition. Tumor size progression was measured at several time points using a caliper, as previously described [12].

### Chronic stress protocol

The restraint stress protocol started a day after the implantation of the L5178Y-R lymphoma. Tumor free- and tumor-bearing mice were individually placed in a restrainer device consisting in a perforated 50 mL conical tube, for 2 h daily over a period of 10 days. Control mice remained in their cages.

### Tissue collection

After completing the 10-day restraint stress protocol, mice were anesthetized with an intraperitoneal injection of ketamine 100 mg/kg and xylazine 10 mg/kg, and then euthanized by cervical dislocation. Tissues were immediately collected, weighed on an analytical balance, and stored at -80 °C until analysis.

### *VEGFR-2* transcriptional expression

RNA isolation, cDNA synthesis, and real-time PCR were performed as previously described [12]. Briefly, RNA was isolated from 30 to 50 mg of tumor, adipose tissue, skeletal muscle and brain samples, using the TRIzol^®^ Reagent (Life Technologies, Carlsbad, CA) according to the manufacturer’s protocol. Isolated RNA was quantified with a NanoDrop 2000 spectrophotometer (Thermo Fisher Scientific, Waltman, MA). Two thousand nanograms of RNA per sample were retrotranscribed to complementary DNA with the High Capacity cDNA Reverse Transcription Kit from Thermo Fisher Scientific according to the manufacturer’s protocol.

For measurement of target *Vegfr2* and glyceraldehyde-3-phosphate dehydrogenase (GAPDH; housekeeping gene for data normalization) genes expression, fluorescence labeled TaqMan probes were acquired from Applied Biosystems, Inc. (Foster City, CA, USA), assay numbers MM01222421_M1 and Mm99999915_g1 respectively. Real-time TaqMan assays were performed with the TaqMan Universal PCR Master Mix, according to the manufacturer’s protocol. Samples were run in duplicate in a 7500 Real-Time PCR System (Applied Biosystems, Waltham, MA, USA). Data were analyzed using the cycle threshold (Ct) method. Comparisons were made using the double Delta CT calculation for each condition as follows: (a) to determine the effect of chronic stress and tumor burden on adipose tissue, skeletal muscle and brain *Vegfr2* expression, levels in resting mouse tissues were selected as calibrator, (b) to determine the effect of chronic stress on tumor *Vegfr2* expression, its expression in tumor of resting tumor-bearing mice was set as calibrator, and (c) to determine the combined effect of chronic stress and tumor burden on relative expression of *Vegfr2* was calculated using the relative expression in tissues of resting, tumor-free animals as calibrator.

### Statistical analysis

Data values for tumor volume, final tumor weight, and inguinal adipose tissue content were expressed as means ± SEM. Differences in adipose tissue content were assessed by analysis of variance (ANOVA) followed by post hoc Tukey testing for pairwise comparisons. Tumor volume and tumor final weight were analyzed with the Student’s t-test. Statistical analyses were performed using the SPSS software (IBM SPSS Statistics Version 21; SPSS Inc., Chicago, IL, USA). *P < 0.05. Transcriptional expression is presented as fold-change relative to the calibrator, calculated using the Double Delta CT method, as described in the Methods section. Differences in fold-change expression were not statistically analyzed.

## Results

**Fig. 1:**
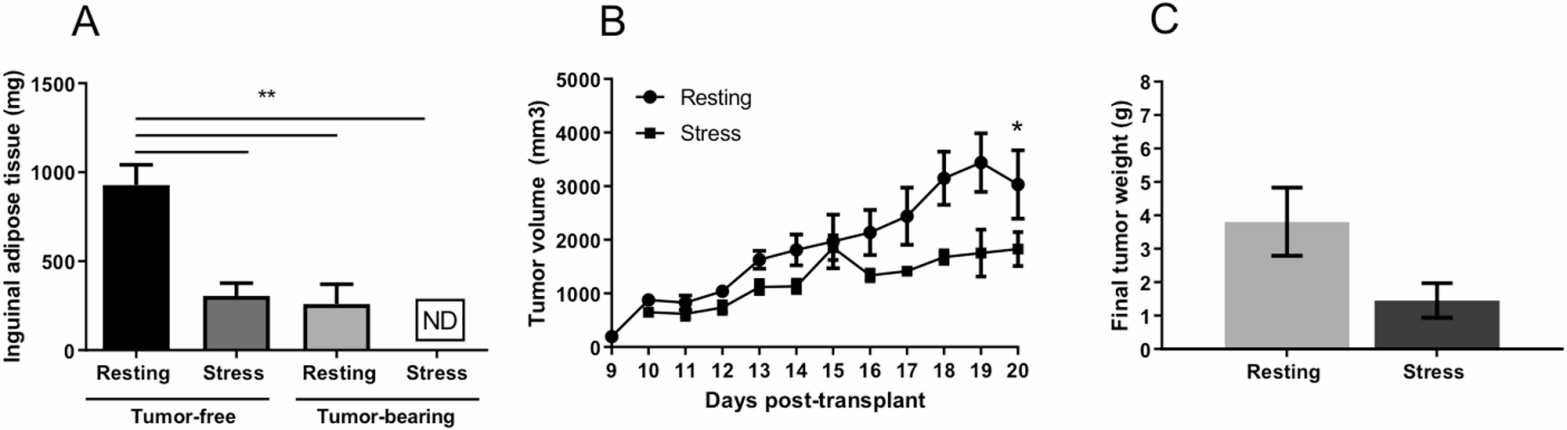
Adipose tissue wasting and L5178Y-R tumor progression in female BALB/c mice subjected to chronic restraint stress. Inguinal adipose tissue content (**A**), tumor volume (**B**), and final tumor weight (**C**) were expressed as means ± SEM. ND: No data. Differences in adipose tissue content were assessed by analysis of variance (ANOVA) followed by post hoc Tukey testing for pairwise comparisons. Tumor volume and tumor final weight were analyzed with the Student’s t-test. *P < 0.05

We assessed the expression of *Vegfr-2* in a mouse model of lymphoma that exhibits features of cachexia, including adipose tissue wasting. In chronically stressed, tumor-free mice, the inguinal fat content decreased significantly (*P* < 0.01) by 67%. Tumor-bearing animals showed a 71% reduction in inguinal fat under resting conditions (*P* < 0.01) (Fig. 1A). Moreover, chronic stress was found to exacerbate adipose tissue depletion in tumor-bearing animals (Fig. 1A). Tumor progression, as measured by volume increase, was delayed under chronic stress (Fig. 1B). In contrast, final tumor weights (Fig. 1C) did not differ significantly between chronically stressed and resting animals (*P* < 0.1).

Regarding *Vegfr2*, its expression increased 6-fold in the adipose tissue of tumor-free animals under stress (Table 1). *Vegfr2* expression was 3-fold higher in the inguinal fat of tumor-bearing animals, as compared with tumor-free animals (Table 1). In contrast, in the adipose tissue of tumor-bearing mice under stress we found that *Vegfr2* expression was 0.02-fold lower (Table 1).

**Table 1:** Changes in the relative expression of *Vegfr-2* in tissues of normal and tumor-bearing BALB/c mice

Experimental conditions				Vegfr-2 expression folds (2^-ΔΔCt)			
#	Restraint stress	Tumor	calibrator condition	Tumor tissue	Adipose tissue	Brain	Skeletal muscle
1	-	-	N/A	N/A	1	1	1
2	+	-	tumor-free, resting	N/A	6.88	10.39	5.65
3	-	+	tumor-free, resting	1	3.07	1.35	3.78
4	+	+	tumor-bearing, resting	10.47	0.02	1.66	6.29
5	+	+	tumor-free, resting	N/A	0.09	1.56	23.81

The relative expression of *Vegfr-2/ Flk1* in tumor, inguinal adipose, brain, and skeletal muscle tissues of mice was quantified using the double Delta CT method. Expression = 1 was assigned to the experimental condition set as calibrator for each comparison of interest as follows: expression in tissues of tumor-free, resting animals to measure the effect of chronic stress (Row 2), expression in tissues of tumor-free, resting animals to measure the effect of tumor burden (Row 3), expression in tissues of tumor-bearing, resting animals to measure the effect of chronic stress in tumor-bearing individuals (Row 4), expression in tissues of tumor-free, resting animals to measure the effect of the co-occurrence of chronic stress and tumor burden (Row 5)

Regarding other tissues, we observed increased *Vegfr2* expression in skeletal muscle from hind limb under chronic stress, a 5.65-fold increase in tumor-free mice, and a 6.29-fold increase in tumor-bearing mice (Table 1). We also observed a 3.78 increase in *Vegfr2* expression in skeletal muscle associated to tumor burdenwhen comparing to muscle expression in tumor-free animals (Table 1). Furthermore, a synergistic effect was observed in skeletal muscle of tumor-bearing mice under stress, where *Vegfr2* expression was 23.81-fold higher when compared with its expression in muscle from tumor-free, resting mice (Table 1).

In brain tissue, a significant 10.39-fold increase in *Vegfr2* expression was found in chronically stressed, tumor-free mice (Table 1). In our study, small increases in *Vegfr2* expression by chronic stress and peripheral tumor burden were found in the brains of lymphoma-bearing mice (Table 1). In tumor, we found up to 10-fold increase in *Vegfr2* expression in animals chronically stressed (Table 1).

## Discussion

We found that *Vegfr2* expression increased in the adipose tissue of tumor-free mice subjected to chronic stress. However, in mice bearing a tumor, chronic stress led to decreased *Vegfr2* expression. In contrast, tumor burden showed to increase *Vegfr2* expression in this tissue. These findings support our hypothesis of the potential of tumor burden to affect *Vegfr2* expression in adipose tissue.

While the effect of chronic stress on adipose tissue angiogenesis and its underlying mechanisms has not been comprehensively investigated, it has been reported that cold-induced chronic stress stimulates adipose angiogenesis through activation of NPY-Y2 receptors [13]. Furthermore, few studies have investigated the effect of stress in VEGFR-2 in adipose tissue. In contrast, the role of adipose tissue vascularization in tumor progression is well recognized [14].

In our study, the content of inguinal fat significantly decreased in resting and chronically stressed tumor-bearing mice (Fig. 1A). Both the stress response and tumor burden are known to induce the remodeling of adipose tissue, acute stress through activation of the sympathetic-adreno-medullar (SAM) axis with secretion of IL-6 [15]. Tumors, on the other hand, can drive fat wasting by secreting molecules such as the ZAG protein, which is implicated in cancer cachexia [16].

We also showed that chronic stress exacerbated adipose tissue loss in tumor-bearing animals, a condition where relative expression of *Vegfr2* was lower when compared to expression in resting animals (Figs. 1A; Table 1). Moreover, the combined effect of chronic stress and tumor burden also decreased *Vegfr2* expression in this tissue (Table 1). Further research is required to address these observations, particularly, the significance of altered *Vegfr2* expression in adipose tissue of tumor-bearing hosts, since VEGFR-2 blockade has been shown to inhibit adipose tissue formation by disrupting adipocyte differentiation [17].

In skeletal muscle from hind limb, chronic stress induced increased expression of *Vegfr2* in both tumor-free and tumor-bearing mice. Tumor burden also induces an increase in *Vegfr2* expression when compared to expression in tumor-free animals. Chronic stress exacerbates the increase in *Vegfr2* expression observed in tumor-bearing mice when comparing with skeletal muscle expression in tumor-free, resting animals. These findings suggests a combined effect of stress and tumor burden in *Vegfr2* expression in skeletal muscle.

In brain tissue, chronic stress induced a significant increase in *Vegfr2* expression of tumor-free mice (Table 1). This finding aligns with previous observations of stress-induced alterations of *Vegfr2* expression changes in the nervous system [8]. Expression of *Vegfr2* has been observed in neuronal cells, where it plays a role in nerve regeneration [18]. In our study, marginal increases in *Vegfr2* expression were found in brain tissue of lymphoma-bearing mice in response to chronic stress and tumor burden (Table 1).

Our study provides evidence of increased *Vegfr2* expression in tumors of mice under chronic stress (Table 1). Increased VEGFR-2 is associated with enhanced tumor progression and poor prognosis [2]. However, in our model, chronic stress slowed tumor progression (Fig. 1B) and did not affect final tumor weight (Fig. 1C). These findings suggest that, in the L5178Y-R lymphoma model under chronic restraint stress, factors other than VEGFR-2 may be more relevant for tumor progression.

## Conclusions

In this study, we provide evidence of increased *Vegfr2* expression in tumor, adipose tissue, skeletal muscle and brain in BALB/c mice under chronic stress. Our findings also indicate that tumor burden affects *Vegfr2* expression in adipose tissue and skeletal muscle. Moreover, tumor burden may synergize with the stress response to enhance the upregulation of *Vegfr-2* on skeletal muscle. Further research is needed to expand on these findings.

### Limitations

Our study has several limitations. First, the small sample size, particularly in the case of adipose tissue, where depletion of this tissue in tumor bearing animals resulted in fewer samples available for gene expression quantification and other analyses. Second, the use of the double delta CT method for calculating relative target expression in multiple tissues and conditions, made impracticable the statistical analysis of relative expression data. Additionally, our findings also require validation at the protein level through *Western Blot* for quantification and/or immunohistochemistry for VEGFR-2 localization in the various tissues analyzed. Furthermore, as only expression in females was assessed, our study did not address the role of sex in *Vegfr-2* expression.

## Data Availability

Raw data and materials are available from the corresponding author at request.

## Abbreviations

VEGF: Vascular Endothelial Growth Factor
VEGFR-2: Vascular endothelial growth factor receptor 2
FLK: Fetal liver kinase 1
KDR: Kinase insert domain receptor
SAM: Sympathetic-adreno-medullar axis
